# Annotations

**Published:** 1901-02-23

**Authors:** 


### ANNOTATIONS.

### Racial and Other Immunity to Fever.

WHEN we obtain the full statistics from India and South Africa in regard to the incidence of typhoid fever on those who have and on those who have not undergone inoculation in the hope of protecting themselves from that disease, we shall be able to speak more positively than is at present possible in regard to the efficacy of that measure. So far as the limited statistics at present available go, they seem to show that inoculation certainly did give some degree of protection. But if typhoid fever is to be studied statistically—and it must be confessed that in a disease of such varying intensity this method is open to manifold sources of error—there are other aspects of the problem which equally deserve careful investigation. Among those who have watched the progress of enteric fever among our troops in India, and have noted the apparent increase in its prevalence which has followed the introduction of the short-service system, the opinion seems to be held that the first year of a soldier's service in India is the dangerous year, and this, if it be a fact—which we believe statistics show to be the case—suggests many thoughts and opens up many lines of inquiry. The explanations commonly given of the excessive incidence of enteric fever during the first two years, and especially during the first year of the Indian tour of service, are: (1) that the young soldier is more careless, and that when he gets to know the ways of the country he learns wisdom; (2) that enteric fever is a disease which falls with especial force upon the young, and that with each year of service the young soldier grows into a less and less susceptible period of life. Both of these explanations may be true so far as they go, and perhaps it is only by a large statistical comparison between military and civil groups at equal ages that anything like a definite answer can be given in regard to their validity. We cannot, however, avoid a suspicion that acclimatisation has something to do with it. Modern theories of immunity are most suggestive in regard to the possibility of people who are long exposed to minor degrees of infection gradually becoming immune to a disease even in its more virulent forms; and when we note the very marked difference in the prevalence of typhoid fever among the British and the native troops in India, and add to that the information given by statistics in regard to the gradually lessening incidence of the disease even upon European troops after a few years of residence in India, we cannot but suspect that much in the same way as a massive inoculation with the typhoid poison is thought to produce immunity, so also continual exposure to degrees of infection insufficient to produce an attack does gradually develop an indifference to the disease itself. At any rate we have to face the fact that we constantly find our troops attacked with this disease notwithstanding all our sanitary precautions, even while all around them are apparently healthy people whose lives are a daily protest against the necessity of taking any precautions at all. People are ready enough to explain this on the hypothesis that it is all due to racial peculiarities; and of course if we are to admit that one race is so built as to be susceptible and another so as to be insusceptible to typhoid infection, all is plain sailing. Recent investigations, however, in regard to the assumed insusceptibility of various dark races to malaria and yellow fever have gone far to knock the bottom out of such easy-going pathology, and it seems highly probable that many of the natives of and long residents in yellow fever districts, who are evidently but slightly susceptible to this disease, are protected, not by virtue of "racial peculiarity," but in consequence of having automatically developed an immunity to the disease from frequent exposure to minute doses of the poison. Whether the same thing happens in regard to enteric fever we cannot say. Perhaps a careful study of the natural history of the Boer, who is said not to be very particular in sanitary matters, might help to elucidate the subject. Whether it is that he hardens himself against thirst and only drinks coffee; or that he is always on the march, and thus does not expose himself to the risks of a crowded life in stationary camps; or that familiarity with dirt has led to a certain degree of immunity to one at least of its consequences, is a matter which has to be decided. It certainly would be a strange, although a not entirely improbable, finale to our long fight against typhoid fever in England if it should turn out that we have produced a population so susceptible to the typhoid poison that our soldiers can only put themselves on a par with the dirt-inured people against whom they have to fight by undergoing a preliminary inoculation with the product of what we have long, and perhaps somewhat arrogantly, held to be a "filth disease."

### The Story of a Dummy Corpse.

THE story of a dummy corpse recently told at the Westminster police court will chiefly appeal to some as being yet another proof that truth is stranger than fiction, and that things are happening day by day in this commonplace London of ours which the novelist would hardly dare to put into his most sensational "penny dreadfuls."

To us the story has another moral, pointing as it does to the very serious deficiencies which exist in the machinery provided for death certification and registration.

That a man should be able to sham Bright's disease by the aid of a little albumen—a thing not difficult to obtain—and should thus impose upon his doctor, is perhaps nothing out of the way. But that this same man, after removing his moustache, should call upon the doctor and inform him of his "poor brother's" death, he and his brother being the same person, and should thus be able to obtain and walk off with his own death certificate, leaving on his bed a dummy corpse to represent his departed self would seem a freak of the imagination had it not all been sworn to in the police court. It was only because the doctor in this case went beyond the legal requirements, and tried to inspect the corpse, that the trick was discovered, and this shows how imperfect is the protection afforded to the public by our present system of registration. Take for example the question of poisoning. We have no hesitation in saying that the existing system of certification of death places but slight difficulty in the way of the ingenious poisoner. The story of the dummy corpse shows how easy it is to get [illegible] certificate, while recent events [illegible] Manchester have shown [illegible] long poisoning if unsuspected may remain undiscovered. In [illegible] of the modern craze for cheap doctoring the machinery for death certification, which is also done "on the cheap," is absolutely out of date. That the certificates which the doctors are forced by law to give for nothing are allowed to be given without their even knowing that their patients are dead will surprise many people, but it is a fact which is recognised and provided for on the certificate forms; while as for burial, although a certificate is always asked for, it is not essential according to law. A body may be interred without any authority whatever, the only proviso being that the person who buries, or performs any funeral service over the body, shall within seven days after the burial give notice thereof in writing to the registrar. All sorts of things may happen in seven days, and even for omitting to give such notice altogether the penalty is only £10. At the present time we trust everything to a mediæval coroner's court, a court whose verdicts are often ridiculous and contemptible. Meanwhile modern methods of insurance put great temptations in men's way; modern methods of cheap and wholesale doctoring make detection of crime unlikely; and modern social habits, which cause neighbours to know but little of each other, greatly lessen the chance of suspicions coming to the coroner's ears. The whole system of death certification requires modernising, and the Government ought to take the matter in hand without delay.

### Propriety in Small Things.

LAST week a case occurred in the Divorce Court in which a medical man was made co-respondent. The plaintiff was evidently an extremely jealous husband, and after the cross-examination of the co-respondent the judge stopped the case and dismissed the petition, saying that the doctor left the court without any stain on his honour or suggestion against his character; which was a very satisfactory end to a very disagreeable accusation. What makes us refer to the case is a little bit of evidence which came out in the cross-examination of the lady, to the effect that the doctor had smoked in her bedroom. Why will doctors do such things? A medical man lives on his reputation; he depends on it for his practice and his very bread, and all his prospects in life may be blasted by the mere suggestion of familiarity with his female patients. One would think then that if for no other reason than his own safety he would walk warily while on duty. Yet we find a doctor smoking in a lady's bedroom! No crime, certainly; hardly an impropriety, perhaps, if the lady likes it. But the silliness of it! Why cannot men remember that a consciousness of rectitude is no defence when charges, which the best of us are liable to, are brought against them, and that what one must think of as a guide to conduct is not always what is right, but what looks right in the eyes of that curious animal, the British juryman? In this particular case all was plain, and it fortunately did not matter. But who can doubt that if a case were to hinge on probabilities, as many cases do, evidence that a man had smoked in a lady's bedroom would be taken as evidence confirming an accusation of familiarity between the parties? Let us take warning even from little things.

### A Lesson from Denmark.

SOCIAL reformers lament over the steady inrush of our working population to the towns, and wonder how it can be checked. It is worth while to bring before them the case of a country which has succeeded in creating a reverse movement, [illegible] in persuading people to leave the town and go into the country. [illegible] accomplished this feat, and can, perhaps, show us how to do [illegible] The chief means seems to be a combination of peasant proprietorship with co-operation. Peasant proprietorship alone won't do it. At least in France, where this is the rule, the tendency of population is to move towards the towns. And in such districts of England as Lincolnshire, where small holdings are more numerous than elsewhere, the people still flock towards the towns. For a small farmer or proprietor, with only a few acres, cannot command the machinery necessary to enable him to compete with his neighbour, who has more land, and a greater command of capital. It is here that co-operation comes in. A co-operative steam dairy will give him full value for his milk, and make it into good butter, whereas he, with primitive appliances and probably yet more primitive ideas, would produce a rancid and unmarketable article. A single day of a hired steam reaper will do as much as many days of labour with the scythe. "Union is strength" in farming, as in other things. At present, it is true, our rural population are hardly inclined to adopt new methods. They go on as their fathers did before them; they are more bound by tradition than any other class, and then they wonder that agriculture is depressed, and that we import corn, butter, eggs and bacon. But for this is not their education, or their lack of education, to blame? In Denmark there are nearly a hundred "People's High Schools," only partly subsidised by Government, where young men and women of the working classes, between eighteen and twenty-five years of age can obtain board and education, both general and technical, in winter, when they are comparatively idle, for ten shillings a week. These schools help to raise the standard of intelligence among the rural population, a standard which is, it must be owned, lamentably low in our country. Surely some such experiments are worth trying, even if they only arrested the inrush of our healthy peasantry to the towns. And that by encouraging our people to stay in the country, we might prevent the nation being so dependent as it is at present on foreign countries for its food-supply, is surely a reason of sufficient importance to justify the bringing before the public all possible means to this end.

